# An Unbalanced QPSK-Based Integrated Communication-Ranging System for Distributed Spacecraft Networking

**DOI:** 10.3390/s20205803

**Published:** 2020-10-14

**Authors:** Na Zhao, Qing Chang, Hao Wang, Zhibo Zhang

**Affiliations:** School of Electronic and Information Engineering, Beihang University, Beijing 100191, China; na_zhao@buaa.edu.cn (N.Z.); wanghao1989@buaa.edu.cn (H.W.); zhangzhibo94@buaa.edu.cn (Z.Z.)

**Keywords:** ranging, communication, unbalanced quadrature phase shift keying (UQPSK), tracking telemetering and command (TT&C), distributed spacecraft networking

## Abstract

The spacecraft tracking telemetering and command (TT&C) system plays an essential role in celestial and terrestrial networks, requiring relative ranging and communication, particularly in satellite formation flying networks and distributed spacecraft networks. To achieve precious ranging and high-data-rate communication in a Master/Slave satellite architecture, an integrated communication-ranging system (ICRS) is introduced. ICRS is based on the inter-satellite spread spectrum ranging and spread/non-spread spectrum communication modulated by unbalanced quadrature phase shift keying (UQPSK). In both uplink and downlink, the in-phase (I) branches and the quadrature (Q) branches undertake the tasks of ranging and communication, respectively. In addition, a global navigation satellite system (GNSS) like signal is adopted in I branches for the sake of better ranging accuracy, and binary phase shift keying (BPSK) modulation is employed in Q branches for a higher data rate. Therefore, the key point of the ICRS design is the power resource allocation between two branches via the selection of a suitable power distribution factor (PWDF). Simulation results demonstrate the good performance of the proposed approach in ranging error and bit error rate (BER). In addition, a reasonable PWDF is recommended. Furthermore, the influence of clock offset is also taken into consideration.

## 1. Introduction

Aerospace engineering is expanding rapidly. The requirements of tracking telemetering and command (TT&C) and communication in a distributed spacecraft network, however, are facing increasingly demanding challenges. A growing number of spacecraft and space missions, such as gravity mapping, remote sensing image transmitting, and water resource location, result in a heavy load in space networks. For traditional TT&C and communication (TT&CC) networks, using single large spacecraft will confront the circumstance in which a breakdown of certain parts causes the failure of the whole system. As the single large spacecraft is limited by volume, mass, power constraints, reconfiguration ability, and flexibility, different small satellites are used to undertake multicomponent space assignments, and can work cooperatively as a system or network, thus requiring inter-satellite communication (ISC) and inter-satellite ranging (ISR) [1,2]. ISC refers to information transmission and message exchange among spacecrafts and ISR refers to measuring and evaluating relative distances among spacecrafts in a network [3,4,5,6,7]. The use of small satellite formation flying rather than individual large-scale spacecraft is the future trend in the field of TT&CC networks.

Typical TT&CC networks in the area of small satellite formation flying include NASA’s Space Communication and Navigation (SCaN), Planet Labs flock constellation [8], and the Global Educational Network for Satellite Operations (GENSO: The GENSO project is not operational at present.) in the European Space Agency (ESA). Furthermore, SCaN is composed of the Near Earth Network (NEN), Space Network (SN), and Deep Space Network (DSN), and is an integrated and complete TT&CC network [9].

The requirements of inter-node communication (INC) and inter-satellite ranging (INR) not only appear in distributed spacecraft networking but also remain a challenging issue in unmanned aerial vehicle (UAV) swarms and other self-organized networks. Furthermore, to reduce hardware complexity and save the transmitting power or spectrum, an increasing number of researchers are paying attention to the design of the integrated communication-positioning networks. These methods can be classified into two groups: (i) merging communication functions into positioning or navigation systems [10,11], and (ii) integrating positioning functions into communication systems [12,13], especially in terrestrial wireless communication systems.

For the first category, the integrated communication-positioning systems are motivated by the need for location reporting in navigation systems. The BeiDou Navigation Satellite System (BDS) is an obvious example of this issue, and can provide high-precision positioning, short message telecommunication, and a timing service. To obtain a high data rate, more exploitations of other integrated methods must be undertaken. In the data link layer, it is recommended by the Consultative Committee for Space Data Systems (CCSDS) standards that communication packets and positioning packets are transmitted alternatively, however, the ranging error will be risen when the access delay is unstable. Thus, it is not suitable for high-precision ranging. In the physical layer, surveys such as that conducted by Richard S. Orr showed that Gaussian filtered minimum shift keying plus pseudo-random noise code (GMSK+PN) is a bandwidth-efficient modulation to realize the integration of communication and ranging [10]. A relatively narrow bandwidth, faster secondary lobe attenuation rate, and a constant envelope characteristic facilitate the popularization of GMSK modulation under the circumstances of deep space exploration [14]. However, this method of demodulating the composite signal with GMSK+PN is intricate in computation and hardware realization, and is more suitable for deep space exploration. Therefore, unbalanced quadrature phase shift keying (UQPSK) is another feasible scheme to integrate communication and ranging.

For the second category, the long term evolution (LTE) system is a typical system in which dual- functional communication and positioning can be considered from the perspective of signal fusion. Because the positioning reference signal (PRS) is discontinuous in the time domain, the receiver confronts the dilemma of signal tracking, which is the major factor about the poor ranging precision [15]. To tackle this discontinuity, a time and code division orthogonal frequency division multiplexing (TC-OFDM) positioning signal is proposed to insert positioning signal into the background noise of the communication signal. Thus, dual functions of communication and positioning can be operated continuously. However, interference exists between the communication signal and the ranging signal. Besides, multi-scale non-orthogonal multiple access (MS-NOMA) scheme is another feasible strategy, in which both the communication signal and the ranging signal are modulated by orthogonal frequency division multiplexing (OFDM). The near-far effect of different positioning users can be overcome by using NOMA, and the signal orthogonality can also be guaranteed by using OFDM. However, the scheme has high computational complexity.

Aiming to meet the requirements of the first category, to reduce the cost of the TT&CC network and diminish the burden of the ground segment, we proposed an integrated communication-ranging system (ICRS) in distributed spacecraft networks. Generally speaking, ISC can be completed by different modulation, while ISR can be determined by inter-satellite measurements, such as time-of-arrival (TOA), time-difference-of-arrival (TDOA), round-trip time (RTT), and received signal strength (RSS), which can be obtained from radio signals. Clock offsets among the nodes are nuisances in utilizing TOA measurements. Multiple satellites are required in using TDOA measurements. RSS has limited accuracy in long propagation distance. In asynchronized networks, RTT is a desirable method to solve the problem of clock offset [16]. The propagation time can be estimated by capturing the response frame, then the precision of RTT is restricted because of the limited frame length. However, by using a code early-late loop in the tracking section, the ranging precision of navigation systems can be reduced to the chip level. In this integrated framework, binary phase shift keying (BPSK) modulation is used in ISC, and the GNSS-like signal is used in ISR. Meanwhile, by inserting the previous local distance measurements into the data subframes, the local satellite can ensure that the opposite satellite can obtain its measurements and tracking the receiving signal at the same time. Then, the clock offset can also be estimated as a by-product in our ICRS.

To the best of our knowledge, recent investigators have mainly employed PN codes to spread spectrum in both channels, with a short PN code in the communication channel and a long PN code in the ranging channel. However, in our proposed ICRS, high-speed data bits are transmitted in the Q branch of the downlink without using short PN codes. Then, the transmitting data rate can be adjusted flexibly by using the channel coding with different channel coding efficiency. For simplicity, the channel coding technology is not discussed in this paper, and the communication performance is only evaluated by the bit error rate of BPSK modulation. Therefore, a communication scheme using high rate downlink transmission without a spread spectrum is constructed. Subsequently, the relationship between ranging precision and carrier-to-noise ratio (CNR), the relationship between bit error rate (BER) and signal-to-noise ratio (SNR), and the influence of the PWDFs on the system performance are analyzed.

The outline of the remainder of this paper is as follows. In Section 2, the system design, proposed algorithm, and underlying theory are presented. Simulations of the ranging precision, BER, and clock offset are described in Section 3. Finally, conclusions are presented in Section 4.

## 2. System Design

**Figure 1:**
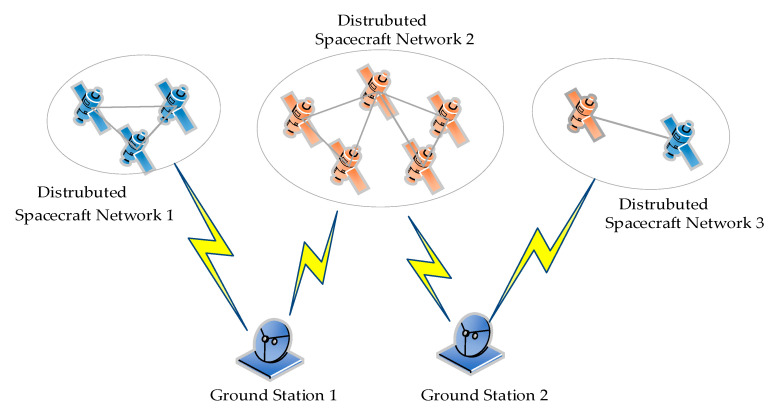
Structure of distributed spacecraft networking.

To realize satellite formation flying autonomously, the proposed scheme is based on a Master/Slave architecture, as shown in Figure 1.

The telecommand instructions and the ranging signals are transmitted from the Master satellite (MS) to the Slave satellite (SS) via uplink, which is operated on the S-band with highly precise ranging and low-speed communication. On the other hand, the ranging signals, telemetry signals, and payloads are transmitted from the SS to MS on via downlink, which is operated on the C-band with high precision ranging and high-speed communication.

In the uplink, the modulation of the two branches is QPSK. In the I branch, the spread spectrum ranging system is designed similarly to a GNSS system, and the signal format in the Q branch is similar to that of the I branch. The only difference between the two links is the data rate. By contrast, in the downlink, the modulation of the two branches is UQPSK. Then, the signal format of the I branch is set identically to that of uplink. However, the Q branch is modulated by BPSK directly. QPSK can be regarded as a special case of UQPSK, in which the power in two branches is equal, then the ICRS can be regarded as being based on UQPSK modulation (for simplicity, QPSK is replaced by UQPSK in the remainder of this paper).

We note that the ranging results are derived from I branches and the demodulated data is obtained from Q branches. In receiver terminals, a series of operations are performed, such as acquisition, tracking, and calculating in the I-branches. Then, to realize the ISC missions, the demodulation in Q branch is accomplished with the aid of the measured Doppler frequency shifts, recovered carrier and clock, which are acquired from the I branch. The signals in the I and Q branches are orthogonal and non-interfering, and the UQPSK signal transmitted in the two links can be modeled as

(1) $$s_{uplink}\left(t\right)=\sqrt{2P_{u}}d_{Iu}\left(t\right)x_{u1}\left(t\right)cos2\pi ft+\sqrt{2P_{u}}d_{Qu}\left(t\right)x_{u2}\left(t\right)sin2\pi ft,$$

(2) $$s_{downlink}\left(t\right)=\sqrt{2P_{Id}}d_{Id}\left(t\right)x_{d}\left(t\right)cos2\pi ft+\sqrt{2P_{Qd}}d_{Qd}\left(t\right)sin2\pi ft,$$

where $P_{u}$, $P_{Id}$ and $P_{Qu}$ are the transmitting signal power in uplink and the transmitting signal power of the two branches in the downlink, respectively; $d_{Iu}(t$), $d_{Qu}\left(t\right)$, $d_{Id}\left(t\right)$, and $d_{Qd}\left(t\right)$ represent the communication signals of two branches in two links, $x_{u1}\left(t\right)$, $x_{u2}\left(t\right)$, and $x_{d}\left(t\right)$ represent the spread spectrum code of the two branches in uplink and I branch in downlink, respectively; and f represents the carrier frequency. In addition, due to the applied UQPSK modulation, the total power is distributed to the I branch and Q branch, and we have

(3) $$P_{Id}=\frac{1}{1+10^{\frac{\alpha }{10}}}P_{d},$$

(4) $$P_{Qd}=\frac{10^{\frac{\alpha }{10}}}{1+10^{\frac{\alpha }{10}}}P_{d},$$

where α represents PWDF and $\alpha =10\mathrm{lg}\left(P_{Qd}/P_{Id}\right)$. α is often chosen as 10 dB in the Tracking and Data Relay Satellite System (TDRSS), and the power of the Q and I branches account for 0.91 and 0.09 of the total power, respectively.

**Figure 2:**
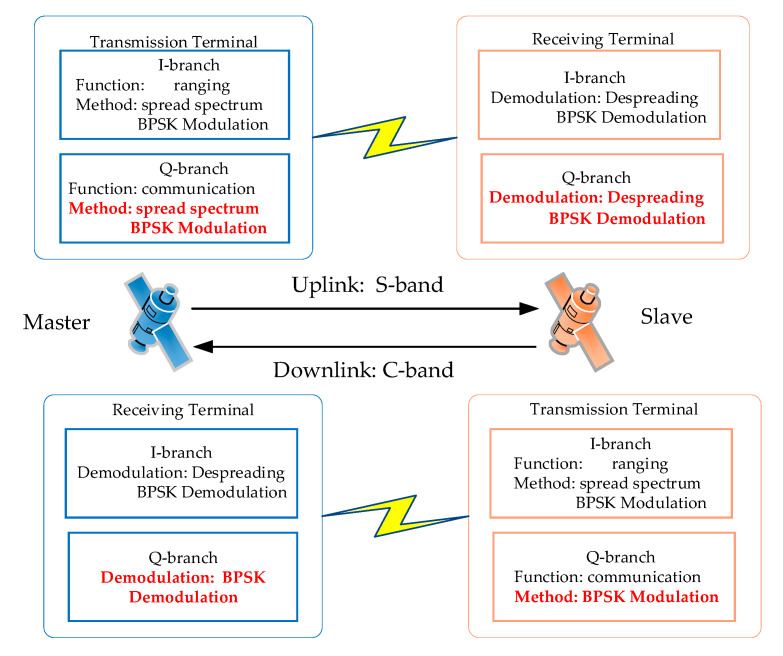
System model of simultaneous ranging and communication in Master/Slave mode.

The primary functions of the UQPSK-based system are summarized in Figure 2 and as follows:

(1) Generating the modulated ranging and communication signals in the I and Q branches;
(2) Receiving, acquiring, tracking the modulated signals, and obtaining the carrier phases and PN code phases from the I branches;
(3) Transferring and demodulating high-speed communication data in the Q branches with the aid of the phase information in the I branches.

### 2.1. Signal Model of Two Links

The signal of the I branches in two links can be described as three parts - transmission data, PN code, and carrier [17]. The carrier frequencies of the two links are on the S-band and C-band. The transmitting data frames in the uplink are modulated by the PN code in both branches, with a PN code rate of 10.23 MHz and a PN code length of 10,230 chips. The transmitting data frames in I the branch of downlink are modulated similar to those of the uplink, with a PN code rate of 20.46 MHz and a PN code length of 20,460 chips. Thus, the symbol period can be calculated as 1 ms.

**Figure 3:**
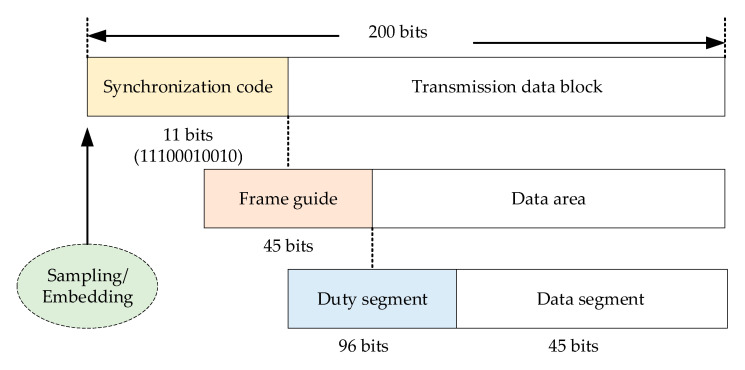
Inter-satellite transmission subframe structure.

Hence, we set the data frame length of I branches of two links are 1000 bits, and each prime frame is divided into five subframes with a length of 200 bits. So when the transmission data frame period of I branch is 1 s, the lasting time of each subframe is 0.2 s. The data fame structure in our system is similar to the CCSDS proximity-1 Version 3 transmission structure. One transmission frame consists of the synchronization code, frame guide, and data area. Furthermore, the front part of data area can be defined as a measuring duty section containing the information about the receiving satellite (opposite satellite) and the remainder of the data area is used to save the transmitting satellite information (local satellite) called a data segment. Figure 3 shows the detailed data structure of each subframe.

The local pseudo-range (LPR) is acquired when the local transmission is synchronized. Then, the dual-way measurement between two satellites is attained by embedding LPR into the duty segment continuously. More details about data transmission are presented in Table 1.

The above subframe structures are employed in both the MS and SS, and data frames are sent periodically between the two. After spreading, the mixture signal is modulated to the carrier, and the Q branch in the downlink is adopted by the non-spreading spectrum BPSK.

### 2.2. Ranging Measurement in Two Links

**Figure 4:**
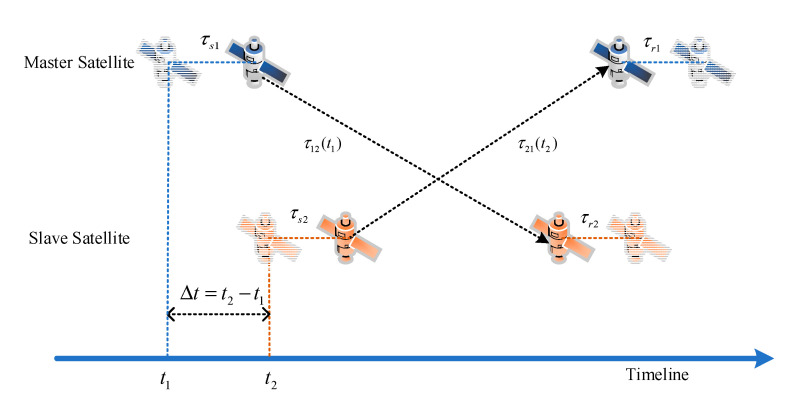
Principle of dual one-way ranging measuring.

The principle of dual one-way ranging (DOWR) measuring based on signal propagation delay in the inter-satellite link (ISL) is shown in Figure 4. The MS and SS simultaneously send GNSS-like signals with their own time tags to each other, and then exchange their own measured pseudo-range and other information, such as the satellite number, subframe number, and local time [18,19].

By receiving the opposite satellite’s signal, two one-way pseudo ranges can be described as

(5) $$\rho_{12}\left(t_{1}\right)=c\cdot \left(-∆t\left(t_{1}\right)+\tau_{s1}+\tau_{12}\left(t_{1}\right)+\tau_{r2}+\varepsilon_{12}\right),$$

(6) $$\rho_{21}\left(t_{2}\right)=c\cdot \left(∆t\left(t_{1}\right)+\tau_{s2}+\tau_{21}\left(t_{2}\right)+\tau_{r1}+\varepsilon_{21}\right),$$

where ∆t is the clock error between the MS and SS, $\tau_{12}\left(t_{1}\right)$ and $\tau_{21}\left(t_{2}\right)$ are the signal propagation delays, and $\tau_{s1}$, $\tau_{s2}$, $\tau_{r1}$, $\tau_{r12}$ refer to the transceiver’s time delay of the MS and SS, respectively. $\varepsilon_{12}$ and $\varepsilon_{21}$ are measurement noises, which contain antenna phase center movement, device delay error, the noise of transmitter and receiver, ionospheric delay error, multipath error, and other noise sources.

The instantaneous relative distance $D_{12}\left(t_{1}\right)$ and instantaneous clock error $∆t\left(t_{1}\right)$ can be calculated by adding and differencing Equations (5) and (6) as

(7) $$D_{12}\left(t_{1}\right)\approx \frac{\rho_{12}\left(t_{1}\right)+\rho_{21}\left(t_{2}\right)}{2}=c\cdot \left[\frac{\tau_{12}\left(t_{1}\right)+\tau_{21}\left(t_{2}\right)}{2}+\frac{\tau_{s1}+\tau_{s2}}{2}+\frac{\tau_{r2}+\tau_{r1}}{2}+\frac{\varepsilon_{12}+\varepsilon_{21}}{2}\right],$$

(8) $$∆t\left(t_{1}\right)=\frac{\rho_{21}\left(t_{2}\right)-\rho_{12}\left(t_{1}\right)}{2c}-\frac{\tau_{21}\left(t_{2}\right)-\tau_{12}\left(t_{1}\right)}{2}-\frac{\tau_{s2}-\tau_{s1}}{2}-\frac{\tau_{r1}-\tau_{r2}}{2}-\frac{\varepsilon_{21}-\varepsilon_{12}}{2}.$$

Hence, differencing Equations (5) and (6) eliminates the clock error between the two satellites, and the instantaneous clock error is calculated by Equation (7), compensation approaches can be employed to mitigate, or even remove the influence of clock error.

### 2.3. Data Flow Demodulation of Q Branch in Downlink

**Figure 5:**
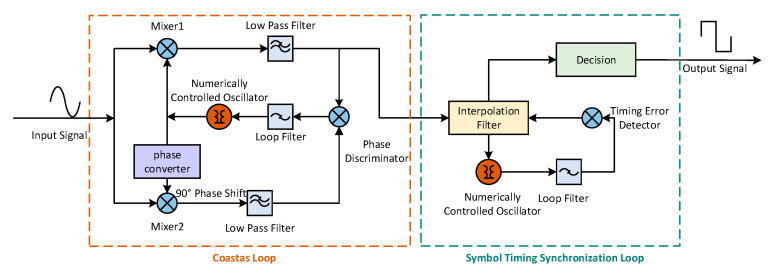
Principle of joint Costas loop and Gardner loop.

The joint Costas loop and Gardner timing loop [20] is adopted to recover carrier and timing information. The local carrier is duplicated by a coherent demodulation method, for example, quadratic loop, decision feedback loop, and Costas loop [21]. The Costas loop is a tracking loop widely used in engineering practices, and the detailed principle is in shown Figure 5.

According to the parameters outlined in the previous subsection, the equivalent CNR of the I branch will increase by approximately 40 dB after despreading, in other words, the spreading gain of the I branch is 40 dB. Thus, more power should be allotted to the Q branch to guarantee the successful demodulation of communication data. Therefore, the input signal in Figure 5 can be expressed as:

(9) $$s\left(t\right)=\sqrt{2P_{Q}}d_{Q}\left(t\right)sin2\pi ft+n\left(t\right),$$

where all parameters are identical to those in Equation (2). Then the outputs of two mixers are

(10) $$s_{I}\left(t\right)=-sin2\pi \hat{f}t\cdot s\left(t\right)=\sqrt{\frac{P_{Q}}{2}}d_{Q}\left(t\right)\cdot \left[cos2\pi \left(f-\hat{f}\right)t-cos2\pi \left(f+\hat{f}\right)t\right]-n\left(t\right)sin2\pi \hat{f}t,$$

and

(11) $$s_{Q}\left(t\right)=cos2\pi \hat{f}t\cdot s\left(t\right)=\sqrt{\frac{P_{Q}}{2}}d_{Q}\left(t\right)\cdot \left[sin2\pi \left(f-\hat{f}\right)t+sin2\pi \left(f+\hat{f}\right)t\right]+n\left(t\right)cos2\pi \hat{f}t,$$

where the parameter $\hat{f}$ is the duplicated carrier through the Costas loop. By using two low pass filters, the noises and high-frequency components can be mitigated to a substantial extent, or even be eliminated, and we have

(12) $${\overset{ˇ}{s}}_{I}\left(t\right)=-sin2\pi \hat{f}t\cdot s\left(t\right)=\sqrt{\frac{P_{Q}}{2}}d_{Q}\left(t\right)cos2\pi \left(f-\hat{f}\right)t,$$

(13) $${\overset{ˇ}{s}}_{Q}\left(t\right)=cos2\pi \hat{f}t\cdot s\left(t\right)=\sqrt{\frac{P_{Q}}{2}}d_{Q}\left(t\right)sin2\pi \left(f-\hat{f}\right)t.$$

The phase discrimination in the Costas loop adopts a two-quadrant arctangent phase detector [22], which is the most accurate detector for the Costas loop, and the result of phase discrimination is

(14) $$∆\varphi =\arctan\frac{{\overset{ˇ}{s}}_{Q}\left(t\right)}{{\overset{ˇ}{s}}_{I}\left(t\right)},$$

where ∆φ represents the difference between the local carrier frequency and the duplicated carrier frequency, which is used to adjust the frequency of the duplicated carrier until the duplicated frequency is almost identical to that of the local carrier. Then, the situation of the tracking loop can be viewed as entering a steady state. Only in this circumstance can the duplicated carrier be regarded as the counterpart of the local carrier, so ∆φ will step into an extremely small value, on the verge of 0 rad, resulting in the Equations (12) and (13) as

(15) $${\overset{ˇ}{s}}_{I}\left(t\right)\approx \sqrt{\frac{P_{Q}}{2}}d_{Q}\left(t\right),$$

(16) $${\overset{ˇ}{s}}_{Q}\left(t\right)\approx 0.$$

We note that ${\overset{ˇ}{s}}_{I}\left(t\right)$ in Equation (15) provides us with an opportunity to realize the data demodulation. In other words, by using the Costas loop, we can not only attain the recovered carrier, but also obtain the demodulated data as a by-product. Because the sampling rate is higher than the data rate, optimal sample points should be chosen from the down-sampling procedure, which aims to obtain the recovered data flow transmitted in every time interval. The symbol timing synchronization loop contains the interpolation filter, numerically controlled oscillator (NCO), timing error detector, loop filter, and decision part [23]. In our proposed system, each transmission time interval has sufficient samples, so that the operation of interpolation is not required to simplify the loop structure. Due to the reasons noted above, the interpolation can be replaced by an iterative shifting linear searching scheme in every transmission time interval. Furthermore, the timing error detector is used to detect the time error from every symbol, and the jitter can be mitigated by the loop filter. This results in the reasonable control of the shift position by the NCO, then the optimal sampling points can be obtained.

With regard to the Gardner algorithm, timing error can be calculated by just two samples. Because the BPSK is adopted in the Q branch, the timing error can be calculated by the Gardner timing error detector as

(17) u(n)=x(nT+T/2){x((n+1)T)−x(nT)},

where u(n) is the timing error, x(nT) is the sample close to the optimal sample, and the other sample requested from the Gardner algorithm is between x(nT) and x((n+1)T). u(n) will be negative if x(nT) pulls ahead of the optimal sample whereas it will be positive if x(nT) falls behind the optimal sample. The real transmission symbol will be recovered after tracking by joining the two loops.

The complete algorithm is summarized in Algorithm 1.

**Algorithm 1:** Joint ranging and communication algorithm in distributed spacecraft networks

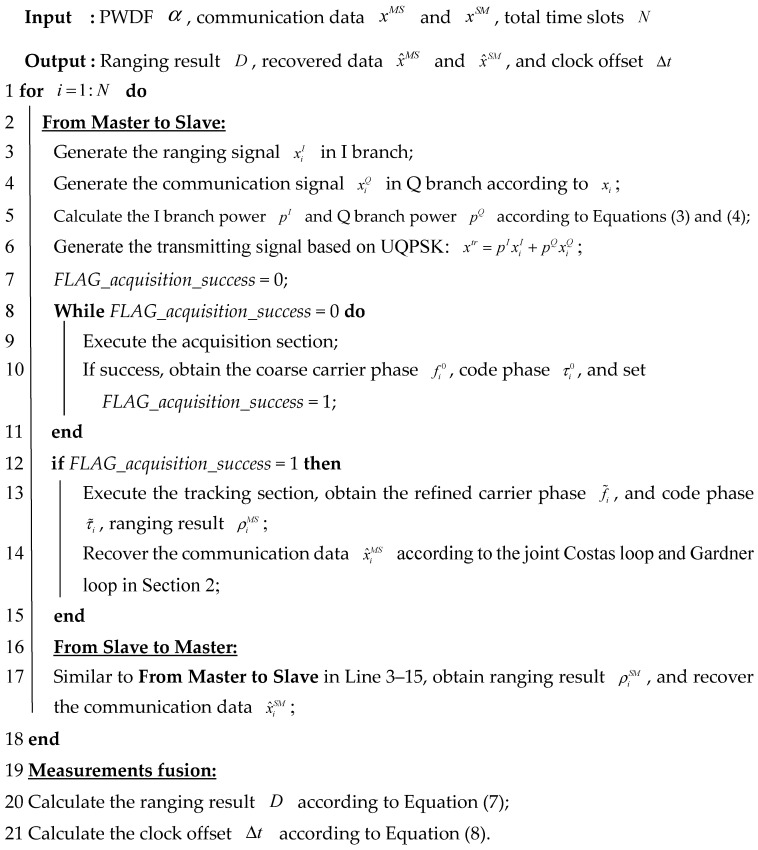



### 2.4. PWDF Versus Ranging Error

The observation of pseudo-ranging is extracted from the tracking loop, without considering the multiple effects and other sources of interferences, and the ranging results are mainly affected by the thermal noise error and the dynamic stress error, which can be described as

(18) $$\sigma_{DLL}=\sigma_{tDLL}+\frac{\theta_{e}}{3},$$

where $\sigma_{tDLL}$ represents the bias of tracking caused by thermal noise, and $\theta_{e}$ represents dynamic stress error of relative motion between the two satellites. In the tracking processing, the dynamic stress error is eliminated with the aid of the carrier loop, and the tracking error can be represented as an empirical formula, calculated approximately as [24,25]:

(19) $$\begin{array}{c} \sigma_{DLL}\\ =\{\begin{array}{c} \sqrt{\frac{B_{L}}{2\cdot C/N_{0}}D\left(1+\frac{2}{\left(2-D\right)T_{coh}\cdot C/N_{0}}\right)},\;\;D\geq \frac{\pi }{B_{fe}T_{c}}\\ \sqrt{\frac{B_{L}}{2\cdot C/N_{0}}\left(\frac{1}{B_{fe}T_{c}}+\frac{B_{fe}T_{c}}{\pi -1}{\left(D-\frac{1}{B_{fe}T_{c}}\right)}^{2}\right)\left(1+\frac{2}{\left(2-D\right)T_{coh}\cdot C/N_{0}}\right)},\;\;\frac{1}{B_{fe}T_{c}}<D<\frac{\pi }{B_{fe}T_{c}}\\ \sqrt{\frac{B_{L}}{2\cdot C/N_{0}}\frac{1}{B_{fe}T_{c}}\left(1+\frac{2}{T_{coh}\cdot C/N_{0}}\right)}.\;\;D\leq \frac{1}{B_{fe}T_{c}} \end{array} \end{array}$$

The parameters in Equation (19) are defined as follows. $B_{fe}$ and $B_{L}$ are RF front-end bandwidth and loop noise bandwidth, respectively. $T_{c}$ is the chip width of pseudo-code and $T_{coh}$ is the coherent integration time. D is correlator spacing and $C/N_{0}$ is the CNR. In our designed system, $B_{L}$, D and $T_{coh}$ are set as 5 Hz, 1 chip and 1 ms, respectively, in both uplink and downlink, and $T_{c}$ is different in uplink and downlink. $T_{c}$ is 1/10,230 ms in uplink, and 1/20,460 ms in downlink. $B_{fe}$ of two links are set as 20.46 and 40.92 MHz, respectively. Based on these parameters, the theoretical ranging error can be calculated according to the middle formula in Equation (19). Therefore, the ranging error under the effect of the PWDF can be derived by substituting Equations (3) and (4) into the middle of Equation (19). The results of $\sigma_{DLL}$ become

(20) $$\sigma_{DLL}=\sqrt{\frac{B_{L}}{2\cdot {\left(C/N_{0}\right)}_{I}}\left(\frac{1}{B_{fe}T_{c}}+\frac{B_{fe}T_{c}}{\pi -1}{\left(D-\frac{1}{B_{fe}T_{c}}\right)}^{2}\right)\left(1+\frac{2}{\left(2-D\right)T_{coh}\cdot {\left(C/N_{0}\right)}_{I}}\right).}$$

The parameter ${\left(C/N_{0}\right)}_{I}$ is regarded as the equivalent CNR of I the branch in two links, which can be summarized as

(21) $${\left(C/N_{0}\right)}_{I_{uplink}}=10\cdot \log10\left(\frac{B_{fe_{uplink}}m_{uplink}}{2}\right)\;\left[dB\cdot Hz\right],$$

(22) $${\left(C/N_{0}\right)}_{I_{downlink}}=10\cdot \log10\left(\frac{B_{fe_{downlink}}m_{downlink}}{\left(1+10^{\alpha /10}\right)}\right)\;\left[dB\cdot Hz\right],$$

where $m=10^{C/N_{0}-10*\log10B_{fe}}$ is the true SNR of the whole mixed signal of the uplink or downlink. The details process of Equations (21) and (22) see the Appendix A.

## 3. Simulation and Analysis

In this section, we simulate and analyze the performance of the designed ICRS in the distributed spacecraft networking algorithm proposed in Section 2, measuring relative distance and exchanging effective messages between the two satellites. All models and simulations were implemented using MATLAB software, and the main components of the receiver include the acquisition, tracking, data demodulation, and symbol timing synchronization part. The acquisition component is similar to signal acquisition in a GPS receiver, which is introduced in [26,27]. Then, the tracking part is also similar to the GPS receiver with a carrier tracking loop and an early-late code tracking loop. Lastly, the data demodulation component is proposed in Section 2.

The ranging error and BER in the two links are two factors that are evaluated in our algorithm, and are related closely to the CNR or SNR in each branch. In the uplink, because of the adoption of QPSK modulation, the transmitting power is distributed to the two branches equally. Therefore, the equivalent SNRs in both of the branches are 3 dB less than that of the total. In the downlink, due to UQPSK modulation, the transmitting power is distributed to the two branches according to the PWDF, resulting in the changes of SNR in two branches. In addition, it is necessary to simulate the clock error between the two satellites, which is determined by DOWR as described in Section 2.

### 3.1. Two Satellites under Synchronous Scenarios

In the synchronous circumstance, we assume that the distance between the MS and SS is 10 km and the two satellites are relatively static, which also can be extended as a relative movement state, and the data rate of uplink and downlink are 20.46 kHz and 20 MHz, respectively. The carrier frequencies of two links are on S-band and C-band, as well as the bandwidths of two links are 20.46 and 40.96 MHz, respectively. We set the CNR in uplink and downlink as 45 and 75 dB·Hz, respectively, and the clock error is set as 0 ns in the simulation. The parameters of the algorithm are listed in Table 2.

#### 3.1.1. Results in Uplink and Analysis

**Figure 6:**
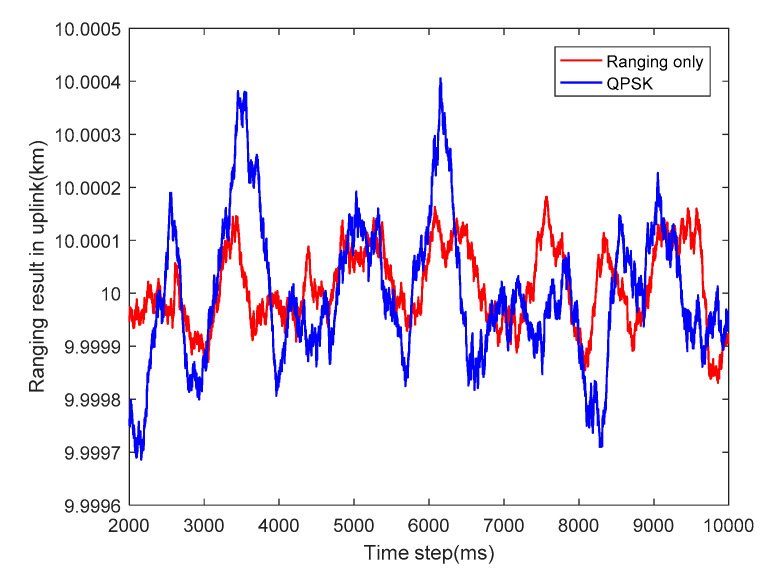
Ranging result in uplink with the ranging branch only and QPSK modulation with ${\left(C/N_{0}\right)}_{uplink}=45$ dB·Hz.

Regardless of other nuisances, the ranging results of PN code ranging in the uplink of the two scenarios with $C/N_{0}=45$ dB·Hz are observed continuously at each epoch depicted, as shown in Figure 6. The ranging error will enlarge after adding a Q branch to communication by occupying half of the total power in the uplink intuitively. Hence, the ranging result of ranging only will have a more violent jitter. Thus, the ranging precision is directly related to the equivalent CNR in the I branch, that is, if we decrease the total CNR or adding a Q branch, the ranging performance also decreases.

#### 3.1.2. Results in Downlink and Analysis

**Figure 7:**
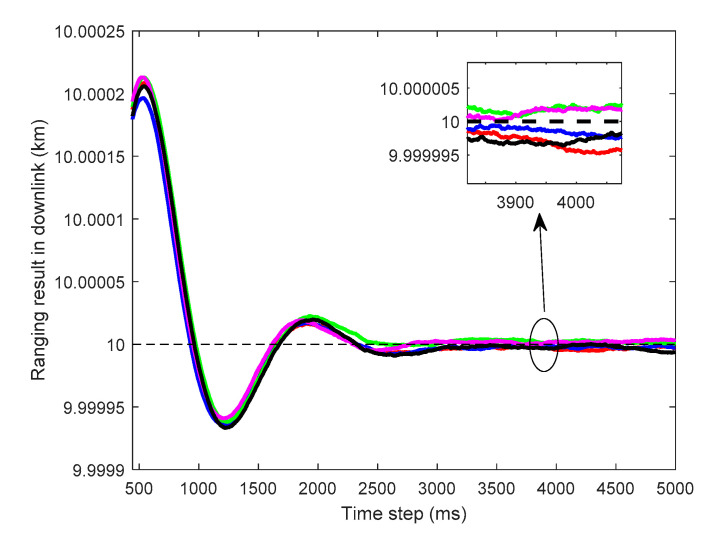
Repetitive experiments about ranging results in downlink.

The ranging results of the I branch in downlink for a given CNR depend on the PWDF and the random noise. Because of the randomness of the noise, the compound of the true signal and the noise can be regarded as a stochastic progress. Therefore, for each execution of the simulation, the received signal can be regarded as a sample function of the stochastic progress, causing the ranging results to fluctuate slightly around the true value. This conclusion can be corroborated by Figure 7, in which ${\left(C/N_{0}\right)}_{downlink}$ equals 75 dB·Hz and the PWDF selected is α=20·dB. The five simulation results form five different curves around the true distance 10 km, and all of the ranging precisions can be accepted.

**Figure 8:**
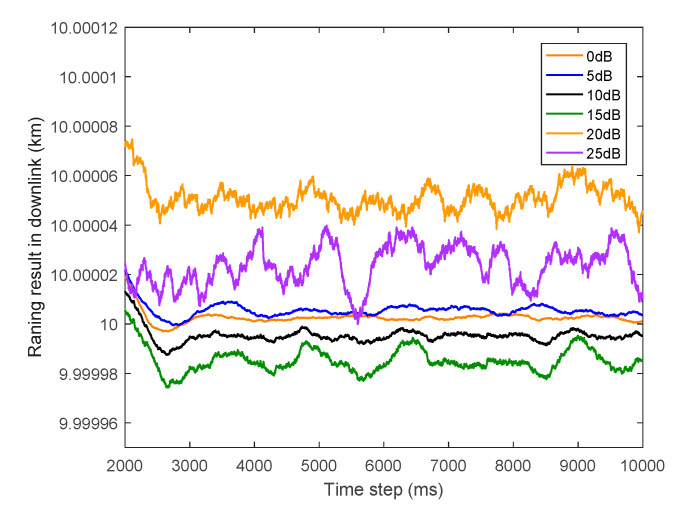
Ranging result in downlink with different PWDFs with ${\left(C/N_{0}\right)}_{downlink}=75$ dB·Hz.

To shed light on the degree of jitter under different PWDFs, Figure 8 shows the ranging results of the proposed algorithm when ${\left(C/N_{0}\right)}_{downlink}=75$ dB·Hz with the PWDF α∈{0,5,10,15,10,25} dB, and the simulation time is set as 10 s. To show the jitter caused by PWDF in detail, the results of the running time from 2 to 10 s are plotted in Figure 8, in which the initial tracking section is omitted. We note that the degree of unsteadiness the ranging results becomes more severe as the PWDF increases. However, this kind of relationship is limited, and the phenomenon becomes insignificant when the PWDF is sufficiently small. For instance, when the PWDFs are 0 and 5 dB, the jitters are relatively close, and their difference is not obviously.

The ranging error is relevant to CNR and PWDF. To evaluate the ranging performance, we execute N=50 Monte Carlo experiments, and calculated and recorded the root mean square error (RMSE) of the ranging error in downlink using Equation (23)

(23) $$RMSE=\sqrt{\frac{1}{N}\overset{N}{\underset{n=1}{\sum }}\overset{T}{\underset{t=1}{\sum }}{\left({\hat{d}}_{t}^{\left(n\right)}-d\right)}^{2}},$$

where ${\hat{d}}_{t}^{\left(n\right)}$ is the corresponding ranging result of the nth trial in the tth time step, and d is the true distance between the MS and SS.

**Figure 9:**
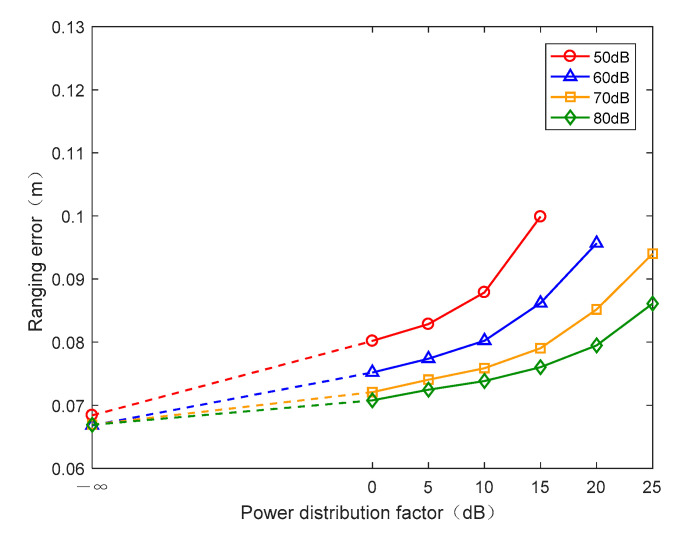
Ranging error in downlink under different CNRs and PWDFs.

According to Equation (22), as the PWDF increases, the equivalent CNR of the I branch in downlink decreases. A lower equivalent CNR of the I branch reduces the ranging precision in downlink. Thus, if the PWDF is negative infinity, the ranging precision reaches its maximum, which can be regarded as a benchmark to evaluate the ranging performance. Then, when the PWDF is 0 dB, the power of the I branch is equal to that of the Q branch. It is obvious that the power of the I branch should be lower, because of the spread spectrum gain resulting from the strong autocorrelation of the PN code. Therefore, the PWDF must be higher than 0 dB in downlink, which ensures the BER of the Q branch is in the range of acceptance. All of the ranging errors in downlink versus different PWDFs and CNRs are illustrated in Figure 9. We note that, when the CNR is 50 dB and the PWDF exceeds 20 dB, the I branch cannot work normally. In addition, when the CNR is 60 dB and the PWDF exceeds 25 dB, the phenomenon is similar to that noted previously. Therefore, it is essential to select a suitable PWDF under various CNRs, or the communication performance cannot be guaranteed.

**Figure 10:**
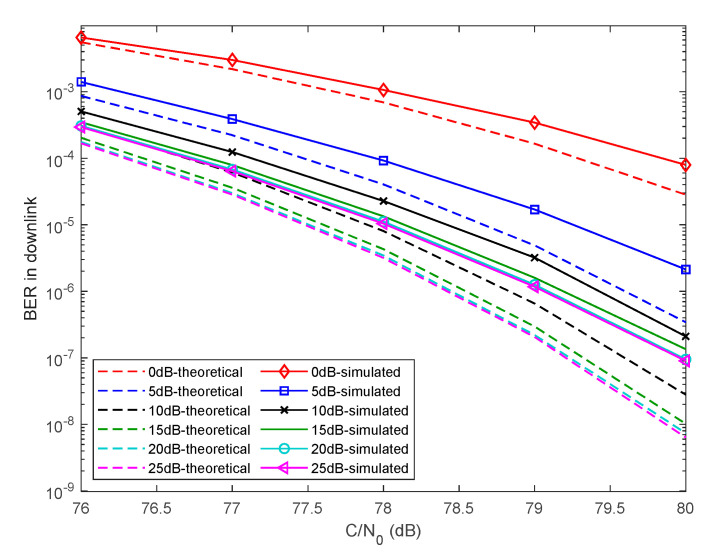
BER of downlink under different PWDF.

With the assistance of the Doppler frequency shift and the code phase acquired from the I branch, the Q branch can be converted to tracking directly, which uses the joint Costas loop and Gardner timing loop to recover local carrier and to realize bit synchronization. Figure 10 shows the BER of the Q branch in downlink versus CNR and PWDF. Both the simulated and the theoretical BERs in downlink versus different PWDFs and CNRs are illustrated in Figure 10. The theoretical BERs are calculated by $(1/2)\cdot erfc\left(\sqrt{SNR}\right)$, where erfc(·) denotes the complementary error function and SNR is transformed from CNR according to the baseband bandwidth. Empirically speaking, the evaluation standard of communication performance, i.e., BER, can be adopted as $10^{-5}$. When the CNR is 80 dB, the BER cannot achieve the standard until the PWDF reaches 5 dB. In addition, it can be seen that increasing α is an efficient but limited means to improve BER. A prominent improvement is achieved when the factor α increases from 0 to 10 dB, but the rate of improvement slows as it increases beyond 15 dB.

In reality, a trade-off exists between the ranging error in the I branch and the BER in the Q branch, thus a reasonable PWDF in downlink should be chosen to balance the performance of the two links. As a suggestion, when the PWDF is selected in the range from 10 to 20 dB, both ranging error and BER behave better and the system performs efficiently.

### 3.2. Two Satellites under Asynchronous Scenarios

**Figure 11:**
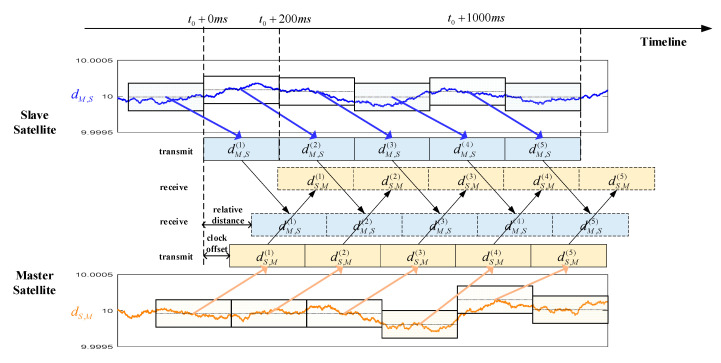
Measured distance exchanging model.

Clock offset is another necessary factor to be considered in a practical system. When the clock offset retains the level of ns, the ranging error will have a bias of at least ten meters. Next, we take the clock offset into our consideration, and give a schematic of the clock offset in a transmission data frame in Figure 11. As introduced in Section 2.1, the transmission data frame period is 1 s, and each subframe lasts for 0.2 s. MS and SS transmit the ranging signal and receive the signal of the other satellite simultaneously. We denote that the distance measurements at MS as $d_{S,M}$, which is derived from the ranging signal transmitted from SS. Similarly, the distances measurements at SS can be recorded as $d_{M,S}$. The local ranging results are depicted as the curves in Figure 11.

For the downlink, we denote the transmission data frame start time of SS to MS is $t_{0}$, then the end time of the first subframe and the first frame is recorded as $t_{0}+200$ ms and $t_{0}+1000$ ms, respectively. When the ith subframe is transmitted from SS to MS, the average distance $d_{S,M}^{\left(i\right)}$ (the mean of ranging results in previous 200 ms) is inserted into its duty segment. A similar process is in uplink simultaneously. Through such a bidirectional process, MS (SS) can receive the average ranging results from SS (MS) and obtain its own local ranging results at the same time. Then, the estimated distances and clock offsets can be calculated from Equations (7) and (8).

**Figure 12:**
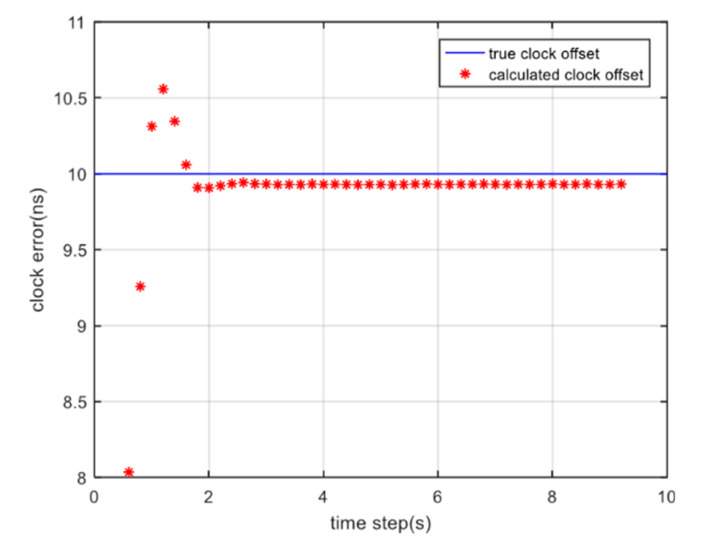
Clock offset between the two satellites.

As described in Section 2.2, we eliminate clock offset as a by-product according to the DOWR algorithm by exchanging timestamps in the two links. The simulation result of the clock offset is shown in Figure 12. The blue line represents the true clock offset which is set as a constant $10^{-9}$ s, and the red asterisk is the calculated clock offset through simultaneous ranging and communication algorithm. In the first two seconds, the tracking loop adjusts to a steady state, thus the calculated clock offset and the true clock offset have an obvious derivation. After the first two seconds, the tracking loop steps into a stable condition, then the calculated clock offset gradually approaches the true value. Through 50 Monte Carlo experiments, the time synchronization precision can be calculated as 0.07 ns. In addition, using Equation (7) we can also calculate the ranging results that are not affected by the clock offset.

## 4. Conclusions

In this paper, a UQPSK-based ICRS in the Master/Slave mode for distributed spacecraft networking is proposed, and the non-interfering ranging and communication are realized stimultaneously. In our scheme, a high-precision ranging GNSS-like signal is adopted in the I branches, while a high-rate communication signal is utilized in the Q branches. By selecting a suitable PWDF, the ranging performance and communication performance are balanced. The simulation results imply that both performances attain an acceptable situation under the condition of the PWDF ranging from 10 to 20 dB, which is in accordance with the factor in the TDRSS selected as 10 dB. When applied to a realistic distributed spacecraft network, UQPSK-based ICRS can directly attach the communication function to the existing navigation system, rather than constructing a new system, which is a promising approach in celestial and terrestrial networks.

## Figures and Tables

**Table 1:** Detail structure of inter-satellite transmission subframe.

Synchronization Code (11 bits)	Frame Guide (48 bits)					Duty Section (96 bits)			
	Satellite ID	Local Time	Subframe Number	Reserved Bits	Opposite Satellite ID	Measuring Opposite Time	Opposite Subframe Number	Local Pseudo Range	Reserved Bits
11100010010	4 bits	32 bits	4 bits	8 bits	4 bits	32 bits	4 bits	48 bits	8 bits

**Table 2:** Basic simulation parameters in simultaneous ranging and communication algorithm.

Category	Parameter	Value
	Sampling frequency	62 MHz
	Intermediate frequency	15.48 MHz
	PN code rate	10.23 MHz
Uplink	PN code length	10,230
(S-band)	Data flow rate in I branch	1 kHz
	Data flow rate in Q branch	20.46 kHz
	Bandwidth	20.46 MHz
	Sampling frequency	320 MHz
	Intermediate frequency	46.52 MHz
Downlink	PN code rate	20.46 MHz
(C-band)	PN code length	20,460
	Data flow rate in I branch	1 kHz
	Data flow rate in Q branch	20 MHz
	Bandwidth	40.96 MHz

## Appendix A. Derivations of Equivalent CNRs in Equations (21) and (22)

We assume the total power ($P_{total}$) of two links is $P_{total_{uplink}}$ and $P_{total_{downlink}}$. Other parameters in uplink and downlink, such as $C/N_{0}$, ${\left(C/N_{0}\right)}_{I}$, m, $B_{fe}$, SNR, $P_{I}$, $P_{Q}$, are denoted similarly.

The equivalent SNR and equivalent CNR in uplink are given by

$$SNR_{I\_uplink}=10\cdot log10\left(\frac{P_{I_{uplink}}}{n_{uplink}}\right)=10\cdot log10\left(\frac{1/2\cdot P_{total_{uplink}}}{P_{total_{uplink}}/m_{uplink}}\right)=10\cdot log10\left(\frac{m_{uplink}}{2}\right),$$

and

$${\left(C/N_{0}\right)}_{I\_uplink}=10\cdot log10\left(\frac{B_{fe_{uplink}}m_{uplink}}{2}\right).$$

Similarly, the equivalent SNR and equivalent CNR in downlink are given by

$$SNR_{I\_downlink}=10\cdot log10\left(\frac{P_{I_{downlink}}}{n_{downlink}}\right)=10\cdot log10\left(\frac{1/\left(1+10^{\alpha /10}\right)\cdot P_{total_{downlink}}}{P_{total_{downlink}}/m_{downlink}}\right)\;=10\cdot log10\left(\frac{m_{downlink}}{\left(1+10^{\alpha /10}\right)}\right),$$

and

$${\left(C/N_{0}\right)}_{I\_downlink}=10\cdot log10\left(\frac{B_{fe_{downlink}}m_{downlink}}{\left(1+10^{\alpha /10}\right)}\right)$$
